# Influence of exercise duration on respiratory function and systemic immunity among healthy, endurance-trained participants exercising in sub-zero conditions

**DOI:** 10.1186/s12931-022-02029-2

**Published:** 2022-05-12

**Authors:** Angelos Gavrielatos, Iluta Ratkevica, Nikolai Stenfors, Helen G. Hanstock

**Affiliations:** 1 Swedish Winter Sports Research Centre, Department of Health Sciences, Mid Sweden University, Östersund, Sweden; 2 Department of Science and Health, Institute of Technology Carlow, Carlow, Ireland; 3 Division of Medicine, Department of Public Health and Clinical Medicine, Umeå University, Umeå, Sweden

**Keywords:** Airway injury, Exercise-induced bronchoconstriction, Cold air exercise, Spirometry, Impulse oscillometry, Airway epithelial damage, Respiratory symptoms, Atopy

## Abstract

**Background:**

Strenuous endurance exercise in sub-zero temperatures can cause airway damage that may lead to EIB. Prolonged exercise can also elicit greater immune perturbations than short-duration exercise. However, the influence of exercise duration on lung function and systemic immunity in sub-zero temperatures has not been established. Additionally, it is currently unknown whether atopic disposition, which is risk factor for EIB, influences respiratory responses in a sub-zero climate. The aim of this study was to compare respiratory and systemic immune responses to two cold air running trials of short and long duration, as well as to examine whether the responses differed between atopic and non-atopic subjects.

**Methods:**

Eighteen healthy, endurance-trained subjects (males/females: 14/4; age: 29.4 ± 5.9 years old; BMI: 23.1 ± 1.7; atopic/non-atopic: 10/8) completed two moderate-intensity climate chamber running trials at − 15 °C, lasting 30 and 90 min, in a randomized, cross-over design. Lung function (spirometry and impulse oscillometry), serum CC16, respiratory symptoms, and blood leukocyte counts were examined before and after the trials.

**Results:**

Lung function was not significantly affected by exercise or exercise duration. CC16 concentration increased after both trials (p = 0.027), but the response did not differ between trials. Respiratory symptom intensity was similar after each trial. There was a greater increase in neutrophils (p < 0.001), and a decrease in eosinophils (p < 0.001) after the 90-min bout. The 90-min protocol increased X5 compared to the 30-min protocol only in atopic subjects (p = 0.015) while atopy increased lower airway symptoms immediately after the 90-min session (p = 0.004).

**Conclusions:**

Our results suggest that a 90-min bout of moderate-intensity exercise at − 15 °C does not cause substantial lung function decrements, airway epithelial damage or respiratory symptoms compared to 30 min running in the same environment, despite a heightened redistribution of white blood cells. However, exercise at − 15 °C may cause airway injury and evoke respiratory symptoms, even at moderate intensity. Atopic status may lead to greater peripheral bronchodilation and higher frequency of respiratory symptoms after long-duration exercise in cold.

*Trial registration:* 01/02/2022 ISRCTN13977758. This trial was retrospectively registered upon submission to satisfy journal guidelines. The authors had not initially registered the study, as the intervention was considered to be a controlled simulation of exercise in a naturally occurring environment (i.e. sub-zero air) for healthy volunteers.

**Supplementary Information:**

The online version contains supplementary material available at 10.1186/s12931-022-02029-2.

## Introduction

Strenuous endurance exercise can induce airway injury that is followed by a restorative process [1–3]. The repeated cycle of injury and repair can trigger the release of pro-inflammatory mediators, disruption of the airway epithelial barrier, and plasma exudation [1, 2, 4], which gradually alter the contractile properties of the bronchial smooth muscle and give rise to airway hyperresponsiveness (AHR), exercise-induced bronchoconstriction (EIB), respiratory symptoms, and asthma [1, 2]. These responses have been collectively termed lower airway dysfunction, and recent report estimates that around 22% of high-level athletes may have some form of lower airway dysfunction [5].

Exercise in sub-zero temperatures, where humidity is low, can present an additional challenge for the airways to condition the inspired air and hence result in greater airway damage and/or obstruction than exercise in warmer conditions [6]. Indeed, the prevalence of lower airway dysfunction in winter athletes is around 1.5 times higher than in summer athletes [5] whilst earlier data reveal that the prevalence of EIB in U.S. Winter Olympians was 23%, but 50% among cross-country skiers alone [7]. Acutely, high-intensity running exercise at − 10 °C, − 15 °C, and − 20 °C results in greater lung function decrements compared to similar exercise in temperatures ranging from 0 to 22 °C [8–10]. However, there remains a lack of evidence pertaining to how the specific training demands of different sports predispose winter athletes to airway damage, and an outstanding need for training strategies that mitigate the risk of developing and triggering EIB. Improved knowledge of airway responses to exercise in cold would enable coaches and athletes to make informed decisions and adjust their training regimes correspondingly.

Exercise intensity has been highlighted as an important factor in determining respiratory responses to exercise in cold environments. High-intensity exercise in sub-zero temperatures has been reported to impair lung function in healthy individuals as indicated by declines in forced expiratory volume in 1 s (FEV1) [8–10] and onset of respiratory symptoms [9, 10]. High-intensity exercise may, however, compromise lung function and cause epithelial damage even in temperatures above 0 °C, since reductions in FEV1 and increased leakage of Clara cell secretory protein 16 (CC16) into blood or urine have been observed shortly after the cessation of training at 4 °C, 9.8 °C and 20.5 °C [11–13]. In addition, interval exercise, where high ventilatory rates are maintained for shorter periods, affects lung function to a lesser extent and elicits less epithelial damage than continuous exercise [14, 15]. Meanwhile, physical activity of moderate-intensity, lasting ≤ 30 min, has not been shown to affect lung function among healthy subjects exercising in cold environments [16, 17]. As the injury-repair cycle in the airways is driven by episodes of severe hyperpnoea [1], this discordance in airway responses between high- and moderate-intensity exercise is likely attributed not only to discrepancies in the pulmonary ventilation rate between the two modalities but also the time spent maintaining high rates of ventilation.

Given participation in sports requiring sustained periods of high ventilation has been highlighted as a risk factor for lower airway dysfunction and potentially greater decrements in lung function after acute exercise in the cold [9, 18–21], it is reasonable to hypothesise that increased duration of acute exercise could also exacerbate airway damage. The underlying mechanisms for such a phenomenon could include larger volumes of inspired unconditioned air and/or a gradual increase in ventilation rate due to fatigue associated with prolonged exercise [22]. In addition, prolonged exercise has been suggested to transiently suppress systemic immune function [23]. As the process of airway epithelial repair follows immediately that of epithelial damage [1, 2], post-exercise suppression of systemic immune function could possibly blunt airway epithelial restitution, although evidence for this is scarce [11]. Considering that winter endurance athletes spend a substantial amount of their training time in low- and moderate-intensity training zones [24, 25], often for prolonged durations, it is highly relevant to explore how exercise duration in a cold climate influences respiratory function, and to our knowledge, no studies to date have characterised respiratory responses to prolonged cold air exercise.

Atopy, defined as a tendency to develop allergic diseases, is another risk factor for diagnosis of AHR, EIB and asthma in endurance athletes [26, 27]. Atopy has been reported in 62.5% of 32 EIB-positive Tunisian athletes [28], while Helenius et al. [27] highlighted that in an athletic cohort, the atopic participants presented significantly more often with AHR and physician-diagnosed asthma than their non-atopic counterparts. As potential heterogeneity of responses to cold air exercise between atopic and non-atopic individuals has never been examined, it would also be worthy to investigate whether atopic status is a factor instigating airway injury after exercise in cold.

The primary aim of this study was to compare the effects of two cold air exercise trials of matched moderate-intensity but different duration (30 vs 90 min) on lung function, biochemical signs of airway epithelial damage, intensity and onset of symptoms and systemic immune parameters in healthy participants. We hypothesised that compared to the 30-min trial, the 90-min trial would result in lung function decrements, greater epithelial disruption and more pronounced symptoms. The secondary aim was to compare responses to the two exercise trials between atopic and non-atopic subjects, with the hypothesis that atopic subjects would exhibit more severe bronchoconstriction, symptoms and epithelial damage than their non-atopic peers.

## Methods

### Study design

The study consisted of one preliminary test followed by two experimental trials performed in a randomised, cross-over design. The experimental trials were carried out in a counterbalanced order and on both visits the exercise sessions took place in an environmental chamber set at − 15 °C [29]. The first trial was conducted at least 48 h (range 2–13 d) after the preliminary test while the two cold air trials were separated by at least 72 h (range 3–8 d) to allow sufficient wash-out for inflammatory responses as well as time for airway epithelial restitution to occur. Each participant performed their trials at the same time of the day to avoid potential circadian effects. Lung function, biochemical and cellular responses and symptoms were assessed pre- and post-exercise during each experimental trial. The study was approved by the Swedish Ethical Review Authority (2020-05205). Data collection took place in winter, during January-March; climate data for the test days was obtained from a local weather station [30].

### Participants

Eighteen volunteers were recruited through advertising in social media and word of mouth, and provided written, informed consent to participate in the study. Participants were endurance-trained men (n = 14) and women (n = 4) without asthma or other chronic cardiovascular or inflammatory disease, and non-smokers. Inclusion in the study required endurance training in sub-zero temperatures, including regular prolonged sessions (> 60 min). Nevertheless, participants had never competed or trained at elite level in endurance sports, to reduce the risk of participants having airway remodelling and/or undiagnosed EIB [31]. All participants lived in the same geographic region and therefore experienced the same outdoor ambient conditions before and during the study period. The subjects were asked to refrain from caffeine on the day of the tests and alcohol for 24 h prior to each visit. Participants avoided strenuous exercise for 24 h before the tests and all volunteers had been free from symptoms of respiratory illness for at least 10 days preceding the tests. All participants that were initially included in the study fulfilled the inclusion and exclusion criteria and completed all experimental protocols.

### Preliminary test

After measurement of height and weight, the participants were administered with a Swedish translation of the Allergy Questionnaire for Athletes © (AQUA©) consisting of 25 questions highly specific for allergy prediction [32]. Each question was assigned a score from 0 to 5. An individual was classified as atopic when their total AQUA© score was ≥ 5. Participants were not included based on atopic status; sub-group analysis would be conducted only if there was a roughly even number of participants with and without atopy. Volunteers completed a maximal incremental exercise test on a motorised treadmill (Rodby Innovation AB, Vänge, Sweden) under ambient laboratory conditions (~ 18 °C), to determine their maximal oxygen consumption ($\dot{\text{V}}$˙O2_max_). Following flow and gas calibration, oxygen uptake ($\dot{\text{V}}$O_2_) was measured using the mixing chamber mode of AMIS 2015 (Innovision AS, Odense, Denmark). The test consisted of four 3-min stages at 1% gradient and incrementally increasing speeds, followed by subsequent 1-min stages at increasing gradient until exhaustion. Heart rate (HR) was monitored using a chest strap and a watch (Polar V800, Kempele, Finland). Speed at 60% $\dot{\text{V}}$˙O_2max_ was predicted via a regression analysis based on $\dot{\text{V}}$˙O_2_ and speed during the four 3-min stages of the incremental test. After the incremental test, subjects rested for 20 min and then ran for 15 min at the predicted speed to elicit 60% $\dot{\text{V}}$˙O_2max_ (1% gradient). Speed was adjusted as necessary until recorded $\dot{\text{V}}$˙O_2_ during the 15-min protocol was within ± 1% of the target $\dot{\text{V}}$˙O_2_.

### Experimental trials

**Fig. 1:**
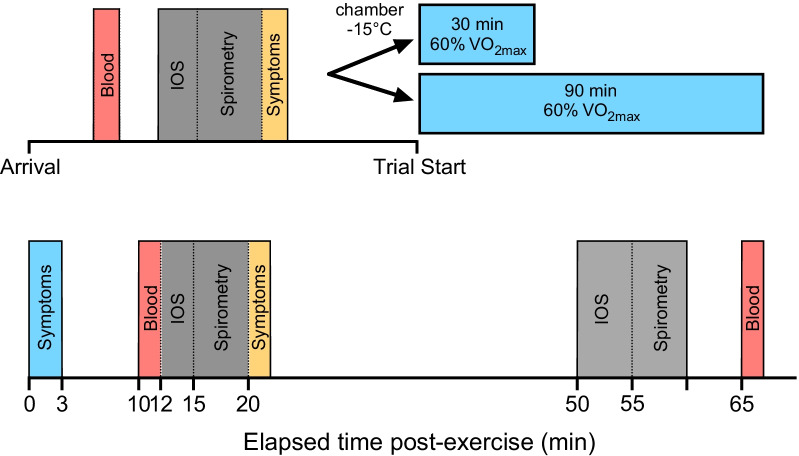
Schematic representation of the experimental trials. Participants remained in the environmental chamber at 0–3 min post-exercise for the symptom reports

On different days, participants performed a 30- and a 90-min treadmill running session in − 15 °C, at a pace corresponding to 60% $\dot{\text{V}}$O_2max_ and a gradient of 1%. HR was obtained every 15 min from which an average value was calculated for each trial. Borg’s 6–20 rating of perceived exertion (RPE) [33] was obtained at the end of the trials. Participants wore the same clothes on both occasions and their nose, mouth and chin remained exposed to the environment to avoid confounding facial cooling [34]. Before and after the running sessions at − 15 °C, the subjects followed a protocol for collection of study variables according to Fig. 1.

### Study variables

#### Lung function parameters

Before, 12 and 50 min after exercise, subjects performed impulse oscillometry (IOS) (Jaeger Vyntus IOS, Carefusion, Germany); 5 reproducible measurements were obtained of respiratory resistance at 5 (R5) and 20 Hz (R20) and reactance at 5 Hz (X5). Before, 15 and 55 min after each trial and immediately after IOS, spirometry was carried out (Jaeger Vyntus Pneumo, Carefusion, Germany) according to the guidelines given by the European Respiratory Society and American Thoracic Society [35]. Participants performed at least 3 efforts within 150 mL of maximum FEV1 and forced vital capacity (FVC), and the values from the best trial were used for analysis. FEV1, FVC and FEV1/FVC were evaluated as the primary outcome measures of spirometry. All measurements for both IOS and spirometry were performed at room temperature by the same trained investigator.

#### Biochemical and cellular markers

Before, 10 and 65 min after the running sessions, blood samples (2 × 3 mL) were collected from the antecubital vein of the participants in EDTA and serum separating vacutainer tubes. EDTA tubes were used for differential cell count analysis of whole blood. Leukocyte, neutrophil, lymphocyte, monocyte, eosinophil, and basophil counts were determined within 10 h of collection (XN-2000, Sysmex, Japan). Blood samples in serum vacutainer tubes were left for 30 min at room temperature to clot and then centrifuged at 2000*g* and 20 °C for 10 min. Serum was then aspirated and stored at − 80 °C until further analysis. CC16 was analysed using Human Clara Cell protein ELISA kits (Biovendor, Modrice, Czech Republic). Mean intra-assay coefficients of variation were < 5% for all the plates. Whole blood and serum biomarkers were adjusted for exercise-induced plasma volume changes using hemoglobin concentration and hematocrit [36].

#### Symptoms

Before, immediately after, and 20 min after each trial, subjects were administered with two questionnaires. The first included a Borg CR10 scale rating the intensity of 9 potential cold-associated symptoms, with 0 reflecting ‘no symptoms at all’ and 10 representing ‘maximal symptoms’ [37]. The symptoms that were assessed involved irritation, and mucus in the nose, irritation in the mouth and throat, cold in the face and the extremities, physical discomfort, irritation in the chest, shortness of breath and warmth in the body. The second questionnaire included a yes/no response regarding the presence of four common lower airway symptoms associated with AHR: cough, wheezing, chest tightness/dyspnea and excessive mucus secretion [9].

### Statistical analysis

Statistical analyses were performed with the Statistical Package for Social Sciences (SPSS) software (Statistics version 27, IBM, Chicago, IL, USA). Normal distribution was assessed using the Shapiro-Wilk test. Data assessed as normally distributed are reported as mean ± standard deviation (SD) or mean and 95% confidence interval (CI) of differences between timepoints or between trials. Non-normal data are reported as median and interquartile range (IQR) and log-normal data as geometric mean and 95% CI. Statistical significance was determined as p < 0.05. To control for type 1 errors, p-values from analyses entailing multiple tests on related variables (baseline characteristics, spirometry, IOS, cell counts and cold-induced symptoms,) were adjusted with the Benjamini-Hochberg (BH) method, using a 5% threshold for false discoveries [38].

Relative percentage changes of IOS and spirometry variables were calculated by dividing the delta (posttrial-pretrial value) by the pretrial value × 100; X5 is presented so that a change towards more negative values is reflected by negative percentage changes. The relative changes were analysed using two-way repeated measures analysis of variance (ANOVA) with trial (30-min and 90-min trials) and time (two post-trial timepoints) as the within-subjects factors. Differences in relative changes of IOS and spirometry parameters between atopic and non-atopic subjects were explored with a 3-factor mixed ANOVA with trial and time as the within-subjects factors and group (atopic and non-atopic) as the between-subjects factor. Paired and independent t-tests were used to compare baseline lung function between the two trials and between atopic and non-atopic subjects, respectively. Bonferroni post-hocs were used to explore significant effects; effect sizes (η^2^_p_) for ANOVA data are also reported where appropriate.

Whole blood cell counts were log-10 transformed before analysis. Due to missing data from missed samples or inadequate quality, linear mixed-effects models (LMM) using the restricted maximum likelihood method were conducted to examine the effect of time and trial on CC16 concentration and leukocyte counts. Trial, time and interaction effects were treated as fixed effects whereas participants as random effects. LMM were also carried out to investigate potential differences in CC16 levels and leukocyte counts between atopic and non-atopic participants. Trial, time, group, and all 2- and 3-way interaction effects were modelled as fixed effects and participants as random effects. Bonferroni post-hocs were conducted where appropriate.

The Wilcoxon-signed ranked test was used to identify differences in symptom intensity between timepoints and trials. The Mann–Whitney U test was performed to examine differences in the intensity of the 9 cold-induced symptoms between atopic and non-atopic subjects. The two-proportion z-test was employed to compare the pooled frequency of occurrence of the four AHR-associated symptoms between trials and time points as well as between atopic and non-atopic individuals. Paired t-tests were performed to investigate differences in HR, RPE, and environmental conditions between the two trials. Independent t-tests were used to compare physical and training characteristics as well as graded exercise test parameters between atopic and non-atopic participants.

## Results

### Participant characteristics, physiological parameters and environmental conditions

Participants’ physical characteristics, graded exercise test parameters, and baseline lung function are presented in Table 1. The average resting baseline FEV1, FVC, R5 and R20 values were within the normal range for age, sex, and height of each participant. Using the AQUA**©**, we identified a positive atopic status in 10 individuals (56%, 7 men, 3 women).

**Table 1:** Physical and training characteristics, graded exercise test parameters, and baseline lung function

	Overall N = 18	Atopic N = 10	Non-Atopic N = 8	p
Age (years)	29 ± 6	29 ± 5	30 ± 8	0.945
Height (cm)	177 ± 9	175 ± 8	180 ± 9	0.172
Body mass (kg)	73 ± 10	72 ± 10	73 ± 10	0.749
Body Mass Index (kg/ m^2^)	23.1 ± 1.7	23.5 ± 1.8	22.5 ± 1.5	0.242
Training hours/year (h)	426 ± 176	444 ± 180	408 ± 180	0.718
$\dot{\text{V}}$O_2max_ (L/min)	4.5 ± 0.8	4.4 ± 0.9	4.6 ± 0.8	0.681
$\dot{\text{V}}$O_2max_ (mL/kg/min)	61.6 ± 8.6	61.2 ± 8.7	62.2 ± 7.2	0.809
HR_max_ (beats/min)	190 ± 8	190 ± 8	189 ± 8	0.740
Speed at 60% $\dot{\text{V}}$˙O_2max_ (km/h)	10.0 ± 1.6	10.1 ± 1.4	9.9 ± 1.4	0.753
$\dot{\text{V}}$E_max_ (L/min)	163 ± 30	158 ± 33	168 ± 26	0.493
$\dot{\text{V}}$˙E at 60% VO_2max_ (L/min)	68 ± 13	67 ± 11	70 ± 15	0.560
FEV1 (%)	105 ± 11	105 ± 11	105 ± 12	0.801
FVC (%)	105 ± 11	102 ± 9	107 ± 13	0.202
FEV1/FVC (%)	81 ± 7	82 ± 6	79 ± 9	0.253
R5 (%)	100 ± 16	102 ± 18	98 ± 12	0.535
R20 (%)	111 ± 16	111 ± 20	111 ± 10	0.953

Besides FEV1/FVC for which ratio values are given, lung function variables are expressed in % of predicted values. Data are reported as mean ± SD. HR_max_: maximal heart rate obtained during the maximal incremental test, $\dot{\text{V}}$˙E_max_: maximal voluntary ventilation achieved during the maximal incremental test, $\dot{\text{V}}$E: ventilation, p: comparison between atopic and non-atopic subjects

Average HR was higher during the 90-min trial (30-min trial: 134 ± 10 beats/min vs 90-min trial: 139 ± 10 beats/min; p < 0.001). Furthermore, post-exercise RPE was higher after the 90-min trial compared to the 30-min trial (11.7 ± 2.1 vs 10.1 ± 1.6; p < 0.001). There were no significant differences in chamber or outdoor environmental conditions between the two trials (Table 2).

**Table 2:** Outdoor and chamber environmental conditions during the two trials

	30-min trial	90-min trial	p
Chamber temperature (°C)	− 14.9 ± 0.0	− 14.8 ± 0.3	0.260
Chamber relative humidity (%)	66 ± 4	68 ± 5	0.149
Chamber absolute humidity (g/m^3^)	1.2 ± 0.1	1.3 ± 0.1	0.068
Outdoor temperature (°C)	− 5.5 ± 5.9	− 4.7 ± 8.5	0.662
Outdoor relative humidity (%)	84 ± 9	82 ± 13	0.690
Outdoor absolute humidity (g/m^3^)	3.0 ± 0.9	3.3 ± 1.4	0.473

Data are mean ± SD

### Lung function parameters

Besides X5, that was significantly more negative before the 90-min trial than before the 30-min trial, baseline lung function parameters did not differ before the two trials (Additional file 1: Table S1). Further analysis revealed that X5 was only significantly more negative before the 90 min trial in the atopic group (Additional file 1: Table S2). There were no differences in the baseline lung function variables between atopic and non-atopic participants (Additional file 1: Table S2).

**Fig. 2:**
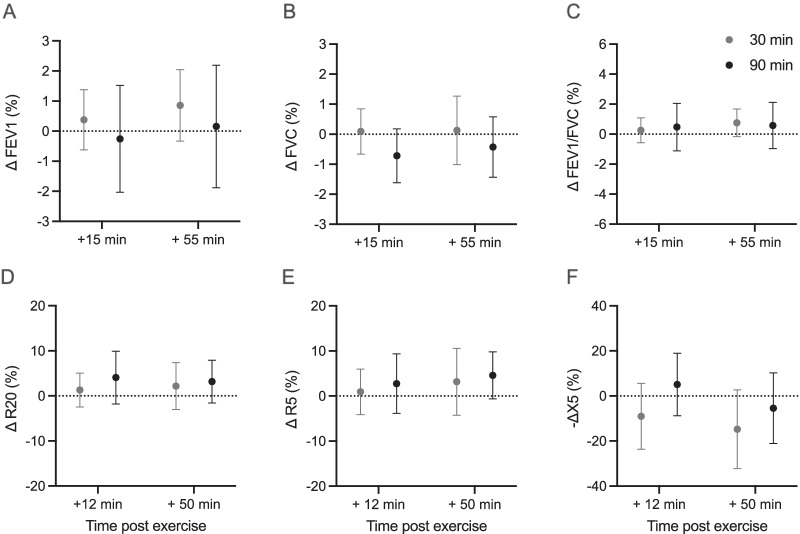
Relative changes in lung function parameters in response to exercise. Data are expressed as % change from baseline values for each trial. Shown are means and 95% confidence intervals of the difference between the baseline and the two post-trial values; Grey: 30-min trial; Black: 90-min trial. For X5, negative change scores represent X5 becoming more negative

**Fig. 3:**
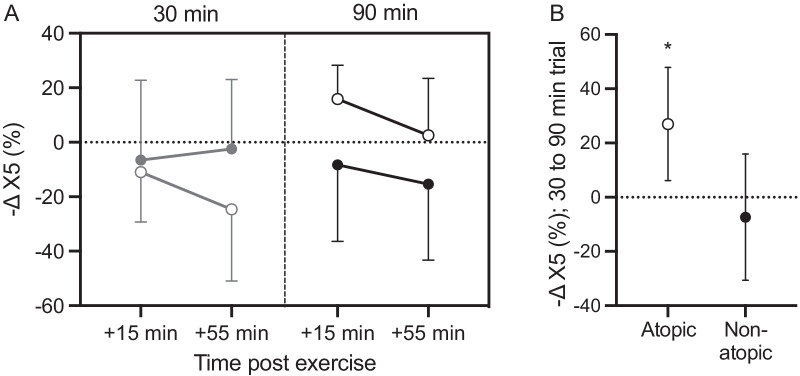
X5 response to exercise in cold, between atopic and non-atopic participants. Open circles represent atopic individuals and closed circles non-atopic participants. Panel **A**: Change in X5 from baseline; data are mean and standard deviation. Grey points and lines represent the 30-min trial and black points the 90-min trial. Panel **B**: Mean difference in X5 between 30- and 90-min trials with 95% CI. *, significant difference between 30-and 90-min trials, p < 0.05

Our analysis did not reveal significant effects of trial, time or interaction effects for any spirometry or IOS variable (Fig. 2). None of the participants experienced EIB post-exercise, defined as a ≥ 10% reduction in FEV1. However, we noticed a significant group × trial (p = 0.015; η^2^_p_ = 0.315) effect for X5 (Fig. 3). In atopic individuals, X5 was less negative after the 90-min trial compared to the 30-min trial, whereas in non-atopic individuals there was no significant difference in X5 between the two trials (p = 0.015; Fig. 3). However, we observed no significant difference in the X5 response to the 90-min trial between the two groups (atopic: 9.2 ± 15.4% vs non-atopic: − 11.9 ± 32.9%; (95% CI: − 3.7 to 45.8); p = 0.09). With respect to atopy, no other significant interaction effects for any other lung function variable were observed (Additional file 1: Table S2).

### Biochemical and cellular markers

**Fig. 4:**
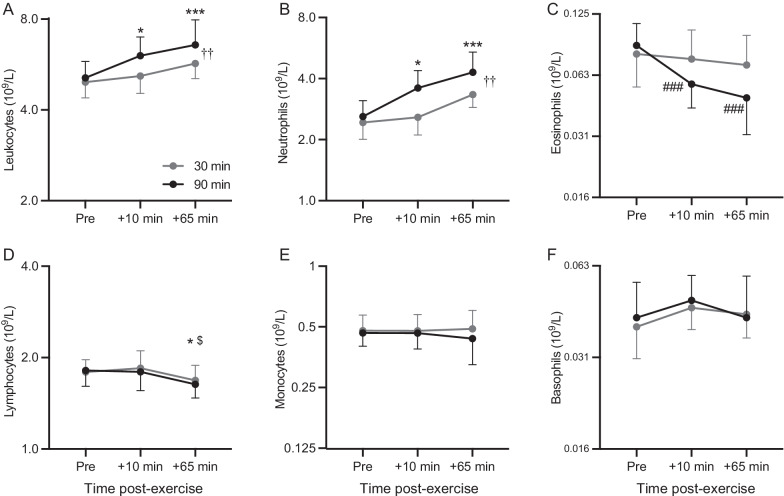
Leukocyte counts before, 10 and 65 min after the 30- and 90-min trials. Grey points and lines indicate 30-min trial and black points and lines 90-min trial. The data were log-normally distributed and are presented using a log_2_ scale on the *y* axis. Displayed are geometric mean and 95% confidence intervals. Significant main effect of time compared to baseline, *p < 0.05, **p < 0.01; ***p < 0.001; $, compared to + 10 min, p < 0.05; Significant difference within trial compared to baseline, ###, p < 0.001; Significant difference between trials, ††, p < 0.01

Both trials induced an increase in leukocyte and neutrophil counts both 10- and 65-min post-exercise (Fig. 4A, B). Leukocytes and neutrophils were also higher during the 90-min trial as indicated by significant effects of trial (Fig. 4A, B). Eosinophils were significantly reduced post-exercise only during the 90-min trial (Fig. 4C). Lymphocytes were significantly lower at 65 min post-exercise compared to before and immediately after exercise on both trials (Fig. 4D). We found no significant effects of trial, time, or interaction effects for monocyte (Fig. 4E) and basophil counts (Fig. 4F). With respect to the atopic and non-atopic groups, there were no significant interaction effects.

**Fig. 5:**
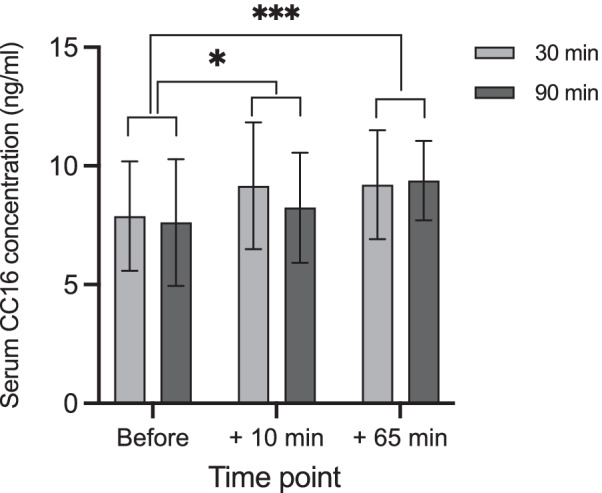
Serum CC16 concentration before, 10 and 65 min after the 30- and 90-min trials. Data are mean and standard deviation. Significant difference from baseline, *p < 0.05; ***p < 0.001

Exercise duration did not influence serum CC16 concentrations. However, CC16 increased 10 and 65 min after exercise in response to both trials (Fig. 5). We observed no significant effects of atopic status on CC16 levels (Additional file 1: Table S3).

### Symptoms

Data on the 9 cold-induced symptoms are presented in Table 3. There were no differences in the intensity of the baseline symptoms between the two trials. Both trials induced significant increases in the intensity of 6 out of 9 symptoms immediately after exercise. Those were nose irritation, production of mucus in the nose, physical discomfort, cold in face, cold in extremities and shortness of breath. All symptoms resolved by 20 min after exercise with the exception of the feeling of a cold face. The sensation of warmth in the body was more intense both immediately and 20 min post-exercise in response to the 90-min trial compared to the 30-min trial (Table 3). The 90-min trial caused a more pronounced feeling of cold in extremities than the 30-min session immediately after exercise while physical discomfort was higher following the 90-min bout when compared to the 30-min session only at 20 min post-exercise. Significant increases in the irritation in the mouth and throat, irritation in the chest and warmth in the body were limited only to the 90 min trial. Finally, there were no differences in the intensity of the 9 symptoms between atopic and non-atopic groups before, immediately after or 20 min post-trials (Additional file 1: Table S4).

**Table 3:** Cold-induced symptoms during each trial

	30 min			90 min		
Symptom	Before	After	+ 20 min	Before	After	+ 20 min
Nasal irritation	0.25 (0–1)	2 (0.25–2)**	0 (0–1)	0.25 (0–1)	2 (0.875–3)**	0.5 (0–1)
Nasal mucus	1 (0.375–2.25)	4 (3–5)***	1 (0.5–2)	1 (0.75–2)	5 (4–6.25)***	0.75 (0.375–2)
Cold face	0 (0–0)	3 (2–4.5)***	0 (0–0.625)*	0 (0–0)	3.5 (3–6)***	0.25 (0–1)*
Cold extremities	0 (0–0.6)	3 (0–4.5)**	0.5 (0–1)	0 (0–0.875)	3.5 (1.75–6.5)**^†^	1.25 (0–3)
Physical discomfort	0 (0–0.5)	2 (0–3)**	0 (0–0.5)	0 (0–0.5)	2.5 (0.5–4)**	0.5 (0–2)^†^
Shortness of breath	0 (0–0.5)	2 (1–2)**	0 (0–1)	0 (0.25–0.5)	2 (1–3)***	0.25 (0–0.5)
Warm in body	1.5 (0–3)	3 (0.75–4)	1 (0.5–3)	2 (0.5–3)	4 (2–5)**^†^	3 (1.25–3.5)^†^
Irritation in the chest	0 (0–0.5)	0 (0–2)	0.25 (0–1)	0 (0–1)	1.25 (0–2)**	0.5 (0–2)
Irritation in the mouth and throat	1 (0.375–2.25)	4 (3–5)	1 (0–2)	1 (0.75–2)	5 (4–6.25)**	1 (0–2.25)

Data are presented as median (IQR). Significant difference within trial, compared to baseline (*, p < 0.05, **, p < 0.01; ***, p < 0.001); ^†^Significant difference between trials (p < 0.05)

There were no significant differences in the frequency of positive answers for the AHR-associated symptoms between the two exercise protocols (Immediately after exercise: 5.9% for the 30-min trial vs 12.5% for the 90-min trial, p = 0.170; 20 min after exercise: 2.8% for the 30-min trial vs 6.9% for the 90-min trial, p = 0.240). Furthermore, neither individual trial elicited significant AHR-associated symptoms immediately or 20 min after exercise, when all participants’ answers were analysed together. However, immediately after the 90-min trial, the frequency of AHR-associated symptoms was significantly higher in atopic than non-atopic participants (22.5% vs 0%, p = 0.004). No significant difference was observed between the two groups by 20 min after the 90 min trial (12.5% in atopic vs 0% in non-atopic, p = 0.038, BH critical value = 0.014). Finally, atopic status did not influence the occurrence of the four symptoms immediately after (10% in atopic vs 0% in non-atopic, p = 0.08) or 20 min after (5% in atopic vs 0% in non-atopic, p = 0.199) the 30-min trial.

## Discussion

We found no differences in post-exercise lung function, serum CC16 levels, and cold-induced and AHR-related symptoms between 30- and 90-min trials of moderate-intensity running at – 15 °C. Nevertheless, the 90-min trial resulted in greater post-exercise neutrophil counts than the 30-min trial. A novelty of this study was that we also examined the potential heterogeneity of responses to exercise at sub-zero temperature in non-asthmatic atopic and non-atopic participants. We found that 90-min running increased X5 compared to 30-min only in atopic subjects. In addition, immediately following 90 min of exercise in the cold, atopic subjects exhibited a higher frequency of AHR-related symptoms than their non-atopic counterparts.

Due to the greater cumulative volume of unconditioned inspired air and an expected gradual increase in ventilation rate during prolonged exercise [22], we hypothesised that the 90-min exercise bout would lead to greater airway dehydration and heat loss, and thus greater declines in FEV1, compared to the 30-min bout. Our results did not support this hypothesis. Although we did not collect data on ventilation during the trials, we observed that average HR was 5 b/min higher and post-exercise RPE 1.6 points higher in response to the 90-min trial compared to the 30-min trial. In addition, immediately after exercise, the feeling of warmth in the body was more pronounced after the 90-min bout, indicating increased exercise-induced thermogenesis. Based on these data, it is reasonable to speculate that the average ventilation rate might have been slightly higher during the 90-min bout but not to such an extent that would lead to more severe airway dehydration and heat loss compared to the shorter protocol. It is important to note that monitoring of oxygen consumption and ventilation rate using standard ergospirometric procedures may have confounded the experimental trials in the present study, since breathing through a mouthpiece or facemask would likely have led to substantial conditioning of inspired air via a heat-exchanger effect. Nevertheless, the physiological and perceptual data obtained during and immediately after the exercise bouts suggest that participants remained in a moderate-intensity exercise zone throughout both trials and that the primary differentiator in exercise stimulus between the two trials was duration, not intensity. Therefore, we conclude that at matched speed, 90 min moderate-intensity exercise in cold does not cause bronchoconstriction compared to 30 min of cold air exercise.

Neither trial elicited bronchoconstriction at 15- or 55-min post-exercise as indicated by no significant changes in FEV1 and FEV1/FVC from baseline. The results from the 30 min trial are consistent with previous evidence that 17 min of walking at − 20 °C did not alter FEV1 [16]. On the other hand, more recent findings revealed that 35 min of moderate-intensity running at − 10 °C decreased FEV1 by approximately 1.7% and FEV1/FVC by 1.6% in healthy participants [37]. The difference in outcomes between the present study and previous results may be attributed to different exercise modalities, intensities and environmental conditions, relatively small response magnitudes, as well as the timing of post-exercise spirometry. As anticipated, our results are also inconsistent with those from studies that have examined the effects of short-duration, high-intensity or exhaustive cold air exercise on spirometric parameters, which typically report 5–7% reductions in FEV1 among healthy individuals [8–10]. As the primary aim of our study was to examine the effect of exercise duration on respiratory health, we selected an intensity that endurance-trained individuals would be able to comfortably maintain for 90 min at − 15 °C. Considering that RPE and HR remained in moderate-intensity zones throughout both exercise bouts, it is unlikely that our protocol would have provoked severe hyperpnoea which is a potent stimulus for bronchoconstriction [1–3, 39]. Furthermore, substantial post-exercise decreases in FEV1 in this context were not necessarily to be expected, given that our participants had no history of asthma or EIB. Our participants also remained in the climate chamber for ∼3 min after cessation of exercise while they completed the two respiratory questionnaires. As there is evidence that inspiration of warm air immediately after exercise in cold may exacerbate bronchoconstriction [39, 40], it is plausible that this delay in chamber exit attenuated airway narrowing. However, there is no consensus regarding how soon an individual should return indoors after an exercise bout in a sub-zero environment. A return to room temperature immediately after exercise in sub-zero climates may not be realistic for most individuals exercising in cold and thus our design typifies real-world settings.

IOS measurements revealed no differences in peripheral airway function at 12 or 50 min post-exercise between the two trials. When obstruction occurs in the lower airways, R5, representing total lung resistance, increases whereas R20, indicating resistance in the intermediate airways, remains unchanged [41]. As R5 and R20 were unaffected by exercise duration, we conclude that the peripheral airways are not more vulnerable to obstruction after 90 min compared to 30 min of exercise at − 15 °C. Furthermore, neither trial increased R20 or R5, which contrasts with previous data reporting a significant increase in R20 in healthy participants following moderate-intensity intermittent exercise (35 min, 62–78% $\dot{\text{V}}$O_2max_) at − 10 °C [37]. The discordance between the two studies may be explained by differences in exercise intensity and session structure as well as to the different seasons in which the studies were conducted [37]. We also measured X5, or respiratory reactance at 5 Hz. X5 reflects the elastic properties of the peripheral airways, and it tends to become more negative in obstructive airway diseases such as asthma and COPD [42, 43]. An acute increase in X5 occurs following pharmacological bronchodilation [44]. In the present study, exercise duration did not influence X5, supporting the conclusion that peripheral airway function does not change following prolonged, moderate-intensity exercise in cold.

Our study also found no post-exercise differences in serum CC16 concentration between the two trials. Contrary to chronic lung diseases, where a progressive decrease in CC16 levels appears to track disease progression [45], acute exercise sessions typically lead to a rise in CC16 concentration [12, 13, 37, 46], that is suggested to protect against inflammation and oxidative stress in the airways, in response to epithelial damage [47]. Exercise-induced hyperpnoea can expose the airway epithelium to dehydration and shear stress [1] which may subsequently increase bronchoalveolar blood barrier permeability and cause leakage of CC16 into the circulation [48]. This may lead to an elevation in circulating CC16 levels that reflects epithelial insult in the distal airways. Our observation that the serum CC16 response did not differ between the two trials corresponds well with the lung function data and illustrates that the 90-min session did not induce greater bronchiolar epithelial disruption compared to the 30-min session. However, both trials elevated CC16 levels at 10- and 65-min after exercise, indicating epithelial injury occurred following moderate-intensity exercise at − 15 °C in both cases. Previous studies have reported increases in plasma/serum CC16 following moderate-intensity exercise in − 10 °C [37] but not room temperature [14]. As inhalation of cold, dry air exerts additional stress on pulmonary epithelial integrity [13], it is likely that even moderate hyperpnoea in a sub-zero climate can cause damage. Thus, although our findings regarding CC16 and lung function responses are not in agreement, we conclude that both exercise trials may have elicited mild airway epithelial damage.

Assessment of common lower airway symptoms did not reveal any differences between the two trials, while among symptoms related to cold air exercise, only the sensations of warmth in the body and cold in extremities were more intense after the 90-min trial. However, these symptoms reflect the thermoregulatory effects of cold air exercise rather than symptoms arising from the respiratory tract. Physical discomfort was also greater after the 90-min trial, but only at 20 min post-exercise. As physical discomfort in our questionnaire represented stiffness in muscle and joints, it is also less informative about respiratory responses. Therefore, the absence of impact of exercise duration on the intensity of respiratory symptoms align with our lung function and CC16 results. Although both trials increased the intensity of respiratory symptoms primarily from the upper airways (i.e., irritation and mucus in the nose) neither trial elicited symptoms associated with lower airway dysfunction. This opposes findings from previous studies employing higher exercise intensities [9, 10]. Considering that high minute ventilation is suggested to determine respiratory symptom onset during exposure to cold [49], these results concur well with current understanding. Based on the collective absence of lower airway symptoms and IOS indicators of distal airway obstruction as well as the transient increase in upper airway symptom intensity, we could suggest that when the cold air reached the small airways, it might have already been well conditioned at the exercise intensity employed in our study. On the contrary, the CC16 increase is not consistent with such a suggestion. Although this highlights the complexity of the underlying mechanisms driving airway damage, it may also be explained by the limitation of a dichotomous questionnaire to detect very mild changes in distal airway function.

Both trials increased blood leukocyte and neutrophil counts 10- and 65-min after exercise and reduced lymphocyte counts 65-min post-exercise. This decrease in lymphocyte counts is consistent with an exercise-induced lymphopenia through a potential redeployment of T-cell subtypes and natural-killer cells [50] while the post-exercise upregulation of leukocyte and neutrophil counts has been attributed to the demargination of leukocytes by vessel walls and/or a catecholamine-mediated mobilisation from hematopoietic tissues [23, 51]. However, the 90-min bout induced a heightened immune cell redistribution as indicated by a decrease in eosinophil counts and a greater increase in neutrophils and leukocytes. Although there are no studies investigating the effect of prolonged exercise in a sub-zero climate on blood leukocytes, our results support the theory that long duration exercise causes a more potent perturbation in the blood leukocyte pool than exercise of short duration [51–53]. Previous work has shown that sputum eosinophils are higher in non-asthmatic competitive cross-country skiers during the winter competitive season, and that cough symptoms were more prevalent during the same time period [54]. Therefore, airway inflammation and reparation seen with non-asthmatic respiratory dysfunction may involve eosinophils. Although highly speculative, the removal of eosinophils from the circulation observed only after the 90-min trial may imply a greater infiltration to the airways. However, this is not in agreement with the absence of differences in CC16 levels, lung function and onset of symptoms between the two trials. Similarly, we are unable to say whether a greater increase in blood neutrophil counts would have translated to a greater influx of neutrophils to the lungs in response to the 90-min trial. Nevertheless, an association between local airway inflammation and neutrophilia may exist, as in vitro data show that exposure of bronchial epithelial cells to cyclic stretch [55] and a hyperosmolar medium [56] stimulates release of the neutrophil chemoattractant IL-8 [11]. Furthermore, activation of bronchial epithelial cells due to osmotic changes in airway surface liquid are followed by secretion of inflammatory mediators and airway neutrophilia [57]. Although the underlying mechanism of the exercise-induced migration of neutrophils into the lungs has not been elucidated, their infiltration to the airways seems to be a secondary event which facilitates the restitution process [11]. As exercise of long duration may cause neutrophils to remain in the blood for up to 6 h [23], such an event could undermine epithelial reparation, as neutrophils would be required in the airways. This would imply that after prolonged training sessions, longer recovery periods may be needed before resuming endurance training. Future investigations aiming to unravel potential associations between systemic immunity and local airway inflammation should incorporate measurement of leukocyte subsets in induced sputum as well as blood.

Our study is the first to examine potential divergent responses to cold air exercise between atopic and non-atopic individuals. While in non-atopic subjects, neither of the trials influenced X5, the atopic individuals experienced an increase in X5 in response to the 90-min trial compared to the 30-min bout, reflecting an increase in lung elasticity due to bronchodilation. However, an important caveat is that X5 was also significantly lower at baseline in the 90-min trial, the reasons for which are unclear. Thus, these results should be interpreted with caution, also considering that the 90-min trial did not affect R5 in atopic volunteers. We also report that immediately after the 90-min trial, the atopic cohort had a significantly higher frequency of lower airway symptoms than non-atopic participants. This is in line with previous data illustrating a significant correlation between atopy and exercise-associated respiratory symptoms [58], but that the difference in lower airway symptoms emerged only after the prolonged exercise bout is a novel and notable finding.

A strength of our study was that our participants were non-asthmatic, AHR/EIB-free and had never competed at elite level. This allowed us to delineate the respiratory responses to prolonged cold air exercise that predispose athletes to EIB or AHR [31] in a population where eventual airway remodelling was less likely to have occurred. Our participants were also a relatively homogeneous group, whose endurance activities involved predominantly running and cross-country skiing. The environmental chamber provided a stable milieu that also simulated environmental conditions met in several circumpolar areas of the world during winter. Moreover, the simultaneous inclusion of respiratory questionnaires, spirometry and IOS as well as the measurement of serum CC16 provided a comprehensive evaluation of responses in the upper and lower airways. Finally, to our knowledge, this study is the first examining how prolonged running exercise in a sub-zero environment affects the blood leukocyte pool.

A limitation of this study was that we were unable to include continuous ventilation measurements during cold air exercise, so as not to interfere with the conditioning of inspired air through a mask or mouthpiece. Furthermore, earlier data have shown that the highest decline in FVC and FEV1 is observed within 5–6 min after exercise with a recovery to baseline levels after around 30 min [10, 59], although FEV1 can remain significantly lower than baseline for up to 20 min post-exercise [17, 60]. However, this is still ambiguous, as studies that have performed repeated spirometry tests within a short time period may have neglected that the deep inhalation-induced bronchodilation effect [61] which is noticed after spirometry may lead to accelerated recovery post-exercise. Nevertheless, as we performed spirometry at 15 min post-exercise, there may have been some recovery before this timepoint so that we did not capture the maximum delta FEV1. Given we observed AHR-related symptoms in the atopic cohort after the 90-min trial but no differences in lung function or CC16 between atopic and non-atopic participants, it is possible that our sample size was too small (10 atopic vs 8 non-atopic subjects) to detect any differences in these outcomes. Finally, the use of control trials also involving 30 and 90 min running in thermoneutral conditions would have provided further information about potential adverse responses to exercise in the cold compared to thermoneutral conditions.

## Conclusions

Our data suggest that for healthy, endurance-trained subjects, a 90-min bout of moderate-intensity exercise at − 15 °C does not cause substantial lung function decrements, airway epithelial damage or respiratory symptoms compared to 30 min running in the same environment, despite a heightened redistribution of white blood cells. That the prolonged training session was not more hazardous to the airways is an important finding for winter endurance athletes, for whom prolonged, moderate-intensity training sessions make up a majority of their training volume. However, both cold air trials elicited signs of airway injury, as indicated by an increase in serum CC16 levels. Therefore, we recommend that individuals exercising in sub-zero climates, even at moderate-intensity, should consider taking precautions to protect the airways if possible, such as using a heat and moisture exchanger [6]. Atopic status may also play a role in the airway response to cold air exercise, as prolonged exercise caused greater frequency of lower airway symptoms in atopic volunteers, as well as more marked bronchodilation in the peripheral airways. Future studies involving comparisons between atopic and non-atopic individuals and exercise at higher intensities are warranted to confirm our findings.

## Supplementary Information

**Additional file 1: Table S1.** Baseline lung function parameters. Data are mean ± SD. Significant p-values are in bold. **Table S2. **Spirometry and IOS results before and after the cold chamber exercise trials in atopic and non-atopic individuals. Data are mean ± SD. *Significant difference from same time point on 30 min trial. **Table S3. **Cell counts and serum CC16 concentration before and after the cold chamber exercise trials in atopic and non-atopic individuals. Data are mean ± SD. No significant interaction effects were found between group and trial or time. **Table S4. **Intensity of the 9 cold-induced symptoms before, immediately after and 20 min after exercise in atopic and non-atopic subjects. Data are presented as median (IR). No significant differences were found between trials or groups.

## Data Availability

The datasets analysed during the current study are not publicly available as participants did not consent to public release of individual data. However, the data are available from the corresponding author on reasonable request.

## Abbreviations

AHR: Airway hyperresponsiveness
ANOVA: Analysis of variance
AQUA©: Allergy Questionnaire for Athletes ©
BH: Benjamini-Hochberg
CC16: Clara cell secretory protein 16
CI: Confidence interval
COPD: Chronic obstructive pulmonary disease
CR10: Category-Ratio 10
EDTA: Ethylenediaminetetraacetic acid
EIB: Exercise-induced bronchoconstriction
ELISA: Enzyme-linked immunosorbent assay
FEV1: Forced expiratory volume in 1 s
FVC: Forced vital capacity
HR: Heart rate
IL-8: Interleukin 8
IOS: Impulse oscillometry
IQR: Interquartile range
LMM: Linear mixed-effect model
R5: Respiratory resistance at 5 Hz
R20: Respiratory resistance at 20 Hz
RPE: Rating of perceived exertion
SD: Standard deviation
$$\dot{\text{V}}$$˙E: Ventilatory rate
$\dot{\text{V}}$˙O_2_: Rate of oxygen uptake
X5: Respiratory reactance at 5 Hz
