# Supplementary material for: Influence of exercise duration on respiratory function and systemic immunity among healthy, endurance-trained participants exercising in sub-zero conditions
Source: Respir Res. 2022 May 12;23:121. doi: 10.1186/s12931-022-02029-2 (PMC9103459; doi:10.1186/s12931-022-02029-2)
Supplement: Supplementary file 1 — Additional file 1: Table S1. Baseline lung function parameters. Data are mean ± SD. Significant p-values are in bold. Table S2. Spirometry and IOS results before and after the cold chamber exercise trials in atopic and non-atopic individuals. Data are mean ± SD. *Significant difference from same time point on 30 min trial. Table S3. Cell counts and serum CC16 concentration before and after the cold chamber exercise trials in atopic and non-atopic individuals. Data are mean ± SD. No significant interaction effects were found between group and trial or time. Table S4. Intensity of the 9 cold-induced symptoms before, immediately after and 20 min after exercise in atopic and non-atopic subjects. Data are presented as median (IR). No significant differences were found between trials or groups. [file 12931_2022_2029_MOESM1_ESM.docx]

**Additional Information**

**Table S1.** Baseline lung function parameters. Data are mean ± SD. Significant p-values are in bold.

|  | 30-min trial | 90-min trial | p | Atopic (n=10) | Non-atopic (n= 8) | p |
| --- | --- | --- | --- | --- | --- | --- |
| FEV1 (L) | 4.638 ± 0.877 | 4.591 ± 0.839 | 0.310 | 4.465 ± 0.861 | 4.802 ± 0.860 | 0.422 |
| FVC (L) | 5.791 ± 1.207 | 5.766 ± 1.233 | 0.413 | 5.421 ± 0.987 | 6.226 ± 1.394 | 0.170 |
| FEV1/FVC | 80.65 ± 7.23 | 80.31 ± 6.56 | 0.631 | 82.40 ± 5.44 | 78.08 ± 7.78 | 0.185 |
| R5 [kPa/(L/s)] | 0.276 ± 0.050 | 0.286 ± 0.047 | 0.282 | 0.288 ± 0.043 | 0.271 ± 0.047 | 0.428 |
| R20 [kPa/(L/s)] | 0.263 ± 0.046 | 0.267 ± 0.044 | 0.567 | 0.271 ± 0.051 | 0.257 ± 0.030 | 0.526 |
| X5 [kPa/(L/s)] | -0.064 ± 0.023 | -0.072 ± 0.024 | **0.006** | -0.069 ± 0.016 | -0.066 ± 0.031 | 0.743 |

**Table S2.** Spirometry and IOS results before and after the cold chamber exercise trials in atopic and non-atopic individuals. Data are mean ± SD. *Significant difference from same time point on 30 min trial.

|  | |  | 30-min trial | | | 90-min trial | | | |
| --- | --- | --- | --- | --- | --- | --- | --- | --- | --- |
|  | |  | Pre | +12-15 min | + 50-55 min | | Pre | +12-15 min | + 50-55 min |
| FEV1 (L) | Atopic | | 4.481 ± 0.890 | 4.505 ± 0.874 | 4.498 ± 0.889 | | 4.450 ± 0.837 | 4.415 ± 0.881 | 4.424 ± 0.886 |
|  | Non-atopic | | 4.835 ± 0.877 | 4.844 ± 0.924 | 4.904 ± 0.884 | | 4.768 ± 0.861 | 4.792 ± 0.839 | 4.823 ± 0.872 |
| FVC (L) | Atopic | | 5.439 ± 1.002 | 5.440 ± 0.978 | 5.413 ± 0.958 | | 5.402 ± 0.975 | 5.371 ± 0.982 | 5.376 ± 1.022 |
|  | Non-atopic | | 6.231 ± 1.360 | 6.238 ± 1.380 | 6.263 ± 1.300 | | 6.221 ± 1.431 | 6.145 ± 1.322 | 6.198 ± 1.383 |
| FEV1/FVC | Atopic | | 82.36 ± 5.75 | 82.80 ± 5.80 | 82.96 ± 5.48 | | 82.43 ± 5.26 | 82.12 ± 6.04 | 82.29 ± 5.47 |
|  | Non-atopic | | 78.51 ± 8.66 | 78.38 ± 8.25 | 79.06 ± 8.59 | | 77.66 ± 7.37 | 78.92 ± 8.62 | 78.82 ± 8.17 |
| R5 [kPa/(L/s)] | Atopic | | 0.282 ± 0.058 | 0.276 ± 0.037 | 0.287 ± 0.047 | | 0.295 ± 0.038 | 0.292 ± 0.054 | 0.298 ± 0.045 |
|  | Non-atopic | | 0.268 ± 0.039 | 0.276 ± 0.055 | 0.275 ± 0.055 | | 0.274 ± 0.057 | 0.295 ± 0.064 | 0.299 ± 0.068 |
| R20 [kPa/(L/s)] | Atopic | | 0.269 ± 0.060 | 0.267 ± 0.045 | 0.273 ± 0.062 | | 0.273 ± 0.046 | 0.278 ± 0.053 | 0.278 ± 0.045 |
|  | Non-atopic | | 0.255 ± 0.020 | 0.262 ± 0.033 | 0.262 ± 0.030 | | 0.260 ± 0.042 | 0.276 ± 0.055 | 0.271 ± 0.051 |
| X5 [kPa/(L/s)] | Atopic | | -0.065 ± 0.015 | -0.070 ± 0.014 | -0.077 ± 0.017 | | -0.074 ± 0.017* | -0.063 ± 0.023 | -0.070 ± 0.022 |
|  | Non-atopic | | -0.063 ± 0.032 | -0.063 ± 0.027 | -0.061 ± 0.024 | | -0.069 ± 0.032 | -0.068 ± 0.019 | -0.073 ± 0.024 |

**Table S3.** Cell counts and serum CC16 concentration before and after the cold chamber exercise trials in atopic and non-atopic individuals. Data are mean ± SD. No significant interaction effects were found between group and trial or time.

|  |  | 30-min trial | | | 90-min trial | | |  |
| --- | --- | --- | --- | --- | --- | --- | --- | --- |
|  |  | Before | +10 min | + 65 min | Before | +10 min | + 65 min | |
| Leukocytes (10^9^/L) | Atopic | 4.70 ± 1.27 | 4.94 ± 1.38 | 5.51 ± 1.36 | 5.25 ± 1.34 | 6.26 ± 1.75 | 7.49 ± 3.16 | |
|  | Non-atopic | 5.49 ± 1.01 | 5.79 ± 1.08 | 6.13 ± 0.99 | 5.27 ± 0.98 | 6.24 ± 1.55 | 5.99 ± 1.08 | |
| Neutrophils (10^9^/L) | Atopic | 2.38 ± 1.21 | 2.48 ± 1.17 | 3.29 ± 1.11 | 2.75 ± 1.11 | 3.81 ± 1.54 | 5.23 ± 2.76 | |
|  | Non-atopic | 2.84 ± 0.72 | 3.06 ± 0.62 | 3.61 ± 0.65 | 2.74 ± 0.52 | 3.87 ± 1.32 | 3.72 ± 0.73 | |
| Eosinophils (10^9^/L) | Atopic | 0.104 ± 0.085 | 0.100 ± 0.086 | 0.091 ± 0.078 | 0.101 ± 0.059 | 0.074 ± 0.070 | 0.079 ± 0.092 | |
|  | Non-atopic | 0.098 ± 0.069 | 0.085 ± 0.066 | 0.081 ± 0.056 | 0.097 ± 0.039 | 0.053 ± 0.018 | 0.042 ± 0.013 | |
| Lymphocytes (10^9^/L) | Atopic | 1.73 ± 0.21 | 1.82 ± 0.35 | 1.61 ± 0.28 | 1.88 ± 0.27 | 1.83 ± 0.47 | 1.65 ± 0.30 | |
|  | Non-atopic | 1.93 ± 0.48 | 1.99 ± 0.61 | 1.83 ± 0.40 | 1.86 ± 0.59 | 1.90 ± 0.68 | 1.67 ± 0.26 | |
| Monocytes (10^9^/L) | Atopic | 0.44 ± 0.15 | 0.45 ± 0.16 | 0.47 ± 0.17 | 0.46 ± 0.12 | 0.47 ± 0.14 | 0.49 ± 0.27 | |
|  | Non-atopic | 0.57 ± 0.12 | 0.57 ± 0.15 | 0.57 ± 0.13 | 0.52 ± 0.15 | 0.52 ± 0.16 | 0.48 ± 0.23 | |
| Basophils (10^9^/L) | Atopic | 0.041 ± 0.018 | 0.047 ± 0.015 | 0.046 ± 0.015 | 0.047 ± 0.016 | 0.054 ± 0.016 | 0.051 ± 0.019 | |
|  | Non-atopic | 0.045 ± 0.015 | 0.049 ± 0.015 | 0.045 ± 0.013 | 0.043 ± 0.017 | 0.045 ± 0.012 | 0.039 ± 0.010 | |
| CC16 (ng/mL) | Atopic | 7.99 ± 2.71 | 9.53 ± 3.29 | 8.83 ± 3.00 | 7.17 ± 30.1 | 7.82 ± 2.49 | 9.44 ± 1.85 | |
|  | Non-atopic | 7.77 ± 1.94 | 8.73 ± 1.87 | 9.60 ± 1.40 | 8.36 ± 1.99 | 8.94 ± 2.00 | 9.29 ± 1.55 | |

**Table S4.** Intensity of the 9 cold-induced symptoms before, immediately after and 20 min after exercise in atopic and non-atopic subjects. Data are presented as median (IR). No significant differences were found between trials or groups.

|  |  | 30-min trial | | | 90-min trial | | |  |
| --- | --- | --- | --- | --- | --- | --- | --- | --- |
|  |  | Before | After | + 20 min | Before | After | + 20 min | |
| Nasal irritation | Atopic | 0.5  (0-1.25) | 2  (0.875-3) | 0.5  (0-1) | 0.75  (0-1) | 2  (1-3) | 0.5  (0.375-1) | |
|  | Non-atopic | 0  (0-0.75) | 0.5  (0-5) | 0  (0-0.375) | 0  (0-0.375) | 1.25  (0-3) | 0  (0-0.375) | |
| Nasal mucus | Atopic | 2  (0.875-2.25) | 4  (3-5) | 1  (0.5-2) | 1.5  (1-2) | 4.5  (4-6) | 1  (0.5-2) | |
|  | Non-atopic | 0.75  (0-2.5) | 4  (3-5.75) | 1.5  (0.25-2.75) | 1  (0-1.75) | 5  (2.25-7) | 0.25  (0-0.875) | |
| Cold face | Atopic | 0  (0-0) | 3  (1.75-4.25) | 0  (0-0.5) | 0  (0-0.125) | 4  (3-6) | 0.25  (0-1) | |
|  | Non-atopic | 0  (0-0) | 2  (3-7) | 0.25  (0-1) | 0  (0-0) | 3  (1.5-6.75) | 0.25  (0-0.875) | |
| Cold extremities | Atopic | 0  (0-1.25) | 3.5  (0-5) | 0  (0-1.25) | 0  (0-0.5) | 4.5  (1.75-8) | 2  (0.375-3.25) | |
|  | Non-atopic | 0  (0-0.875) | 2  (0-5) | 0  (0-0.375) | 0  (0-0.5) | 3.5  (0.25-4.75) | 0.5  (0-0.275) | |
| Physical discomfort | Atopic | 0  (0-0.5) | 2  (0.75-3) | 0.25  (0-0.625) | 0  (0-0.625) | 2.5  (0.375-4.5) | 0  (0.5-2) | |
|  | Non-atopic | 0  (0-0.375) | 0  (1-3) | 0  (0-0) | 0  (0-0.375) | 2.5  (0.125-3.5) | 0.5  (0-1.75) | |
| Shortness of breath | Atopic | 0  (0-0.5) | 2  (0.875-2) | 0  (0-0.625) | 0.5  (0-0.5) | 2  (0.875-3) | 0.5  (0-0.625) | |
|  | Non-atopic | 0  (0-0.75) | 1  (1-3) | 0.25  (0-1) | 0  (0-0.5) | 2  (1-3) | 0  (0-0.5) | |
| Warm in body | Atopic | 2.5  (0-3) | 3  (1.75-4) | 1  (0.5-3) | 1.5  (0.5-3) | 4  (3-4.25) | 2.5  (2-3.25) | |
|  | Non-atopic | 1  (0.125-4.25) | 0  (3-4) | 1.5  (0.125-3) | 2  (0-2.75) | 3.5  (1-5.75) | 1.5  (0-4.5) | |
| Irritation in the chest | Atopic | 0.5  (0-0.625) | 1.5  (0.75-2) | 0.75  (0.5-1.25) | 1  (0-1.25) | 2  (1.5-2.5) | 0.75  (0-2.25) | |
|  | Non-atopic | 0  (0-0) | 0  (0-0) | 0  (0-0) | 0  (0-0.375) | 0  (0-0.5) | 0  (0-1.75) | |
| Irritation in mouth and throat | Atopic | 2  (0.875-2) | 2  (1.75-3) | 1.5  (0.875-2) | 1.5  (1-2) | 2  (2-4) | 1  (0.5-2) | |
|  | Non-atopic | 0  (0-0.875) | 2  (0.5-3) | 0  (0-2) | 1  (0-1.75) | 0.75 (0.125-3) | 0  (0-3) | |
